# Poisson Multi-Bernoulli Filter Driven Information-Controlled Selection of Pose Graph Constraints for SLAM

**DOI:** 10.3390/s26134138

**Published:** 2026-07-01

**Authors:** Tao Li, Ying Hu, Zijing Zhang, Fei Zhang

**Affiliations:** 1 Ocean College, Jiangsu University of Science and Technology, Zhenjiang 212100, China; 2 School of Instrument Science and Engineering, Southeast University, Nanjing 210096, China

**Keywords:** simultaneous localization and mapping, poisson multi-bernoulli filtering, pose graph optimization, real-time SLAM, stochastic finite set

## Abstract

Traditional SLAM methods face significant challenges in complex environments, including high computational complexity, ambiguous data association, and limited real-time performance. Existing approaches often rely on explicit data association or computationally intensive filtering frameworks, which restrict their scalability and robustness. In this paper, we propose a pose-graph-optimization-based Poisson multi-Bernoulli (PMB) SLAM framework. The proposed method models the map as a unified structure consisting of undetected features represented by a Poisson point process (PPP) and detected features represented by multi-Bernoulli (MB) components, enabling consistent feature estimation while reducing the reliance on explicit data association. Furthermore, an information-controlled pose graph constraint selection strategy (IC-PGCS) is developed to effectively couple PMB filtering with pose graph optimization, allowing adaptive activation of graph optimization based on accumulated information. Simulation results demonstrate that the proposed method achieves comparable map feature estimation accuracy while improving computational efficiency and real-time performance compared with RB-PHD-SLAM and multi-Bernoulli-based SLAM methods. These results validate the effectiveness of the proposed framework for SLAM applications in cluttered indoor environments.

## 1. Introduction

Simultaneous Localization and Mapping (SLAM) is one of the core technologies enabling autonomous navigation in mobile robots. With advancements in navigation and perception technologies, mobile robots are increasingly deployed in complex environments such as indoor fire rescue operations and dusty workshop inspections—scenarios characterized by low signal-to-noise ratios, strong noise interference, and high susceptibility to detection failures. This demands higher standards for positioning accuracy and real-time performance in complex scenarios. Balancing computational efficiency and system stability while maintaining accuracy has become a significant research challenge in the SLAM field [1,2,3,4,5,6,7,8]. Existing SLAM methods can generally be categorized into two major types: filter-based SLAM methods and optimization-based SLAM methods [9,10]. Among these, filter-based methods, represented by Kalman filters and their extended variants, often suffer from inaccurate data association and reduced estimation accuracy in complex indoor environments due to difficulties in data association, accompanied by significant computational overhead. To circumvent explicit data association challenges, Mahler R [11,12,13] introduced the Random Finite Set (RFS) theory, which subsequently inspired the development of the Probabilistic Hypothesis Density (PHD) filter [11], the Conditional Probabilistic Hypothesis Density (CPHD) filter [12], and the Multi-target Multi-Bernoulli (MeMBer) filter [13]. Subsequently, Vo and his team addressed engineering application needs by implementing Gaussian Mixture (GM) and Sequential Monte Carlo (SMC) versions of PHD and CPHD, proposing algorithms such as GM-PHD, GM-CPHD, SMC-PHD, and SMC-CPHD [14,15] to achieve multi-target tracking in both linear and nonlinear environments. Additionally, Vo proposed the Cardinality Balanced MeMBer (CBMeMBer) filter [16], which incorporates the idea of cardinality balancing into the MeMBer architecture and a portion addresses the issue of overestimating the number of targets. Adams M et al. [17] proposed a novel feature generation strategy that incorporates the previous observation set into the current prior information for PHD updates. However, due to limited prior information sources, this approach still suffers from insufficient accuracy in estimating map feature locations and quantities. In 2018, Fernandez, Williams, and colleagues proposed the Poisson Multi-Bernoulli Mixture (PMBM) filtering algorithm [18], demonstrating its conjugate prior property and presenting its GM implementation mode, thereby introducing the GM-PMBM filtering algorithm. This algorithm divides the target state into two components: a Poisson point process and a Multi-Bernoulli Mixture (MBM). During tracking, only the parameters of these two components need to be passed to estimate the target state, resulting in high computational efficiency. Subsequently, considering the tracking requirements of derived targets, Svensson et al. applied the PMBM filtering algorithm to tree-like trajectory sets, proposing the TrPMBM filtering algorithm [19,20,21]. Although the PMBM filtering algorithm demonstrates excellent accuracy in multi-target tracking, its high computational complexity leads to significantly increased processing time in complex scenarios. To address this issue and enhance tracking real-time performance, Xia Yuxuan et al. proposed the Poisson Multi-Bernoulli (PMB) filtering algorithm [22] based on the PMBM approach. This algorithm substantially reduces computational complexity by approximating the MBM term as a single Multi-Bernoulli (MB) term. While this approximation strategy sacrifices some precision, it represents an acceptable trade-off in many practical applications where rapid processing of numerous targets while maintaining satisfactory tracking performance is paramount. Grisetti et al. [23] proposed a pose graph-based SLAM method, but its inability to produce real-time results limits its application in dynamic scenes. Thrun et al. [24] introduced a sparse extended Kalman filter SLAM approach combining EKF with graph optimization principles to enable online estimation, yet it still struggles to effectively handle target consistency and data association issues in complex environments. Zhang Q Y et al. [25] proposed a pose graph-optimized particle filter SLAM method, which partially alleviated particle degeneration issues. However, its reliance on the particle filter framework resulted in significantly increased computational load in high-noise and high-feature-density scenarios. Recently, DYGS-SLAM [26] has focused on realistic map reconstruction in dynamic scenes using double-constrained visual SLAM, which is complementary to our radar-based approach in static cluttered environments. Meanwhile, graph-based SLAM has been extended to exploration tasks with prior topo-metric information to enhance pose graph reliability [27]. In the RFS-SLAM domain, set-type belief propagation has been applied to PMB-SLAM as an alternative to filtering-based implementations [28].

Compared to GB-CBMber-SLAM, which relies on a cardinality-balanced multi-Bernoulli filter with AIC-triggered graph optimization, our method adopts a Poisson multi-Bernoulli (PMB) representation that explicitly separates undetected features (PPP) from detected ones (MB), reducing the reliance on explicit data association. Our information-controlled constraint selection (IC-PGCS) uses a heuristic score based on motion edges and observed features rather than an AIC criterion, and is integrated into the PMB update loop. This design addresses the limitations of traditional RFS-based SLAM methods in complex indoor environments—such as reduced estimation accuracy caused by first-order approximations (e.g., PHD) in map inference, as well as the high computational cost and limited real-time performance of particle filter-based pose estimation—leading to the proposed Graph-Based Poisson Multi-Bernoulli SLAM framework (GB-PMB-SLAM).

The main contributions of this work are summarized as follows:

(1) PMB-based map estimation with enhanced uncertainty modeling. A Poisson Multi-Bernoulli (PMB) filtering framework is introduced into the SLAM map estimation process, where the map is modeled as a hybrid Random Finite Set (RFS) consisting of Poisson and multi-Bernoulli components. This formulation enables the modeling of false alarms, missed detections, and feature birth/death processes within a unified probabilistic framework, improving the robustness of feature cardinality estimation and posterior state inference in cluttered environments.

(2) Pose graph optimization for improved efficiency and consistency. Pose graph optimization is incorporated into the SLAM framework to replace traditional Rao–Blackwellized particle filtering for pose estimation. Motion constraints and observation constraints derived from map feature posterior distributions are integrated into a graph-based optimization formulation, which helps reduce sampling overhead while improving global consistency and computational efficiency.

(3) Information-guided coupling between map and pose estimation. A connection mechanism between PMB filtering and pose graph optimization is developed by constructing observation constraints based on posterior relationships of map features across multiple time steps, which reduces the reliance on explicit data association. In addition, an information-control score-based pose graph constraint selection strategy (IC-PGCS) is introduced to evaluate constraint quality and select reliable constraints for feedback into subsequent map updates. This design supports a more effective interaction between map estimation and pose optimization, contributing to improved robustness and efficiency.

Extensive experiments under high-noise and cluttered indoor environments with moderate feature density show that the proposed method achieves competitive performance compared with representative RFS-based SLAM approaches, including RB-PHD-SLAM, MBer-SLAM, and GB-CBMber-SLAM [29], with improvements in localization accuracy, map stability, and computational efficiency.

## 2. Materials and Methods

### 2.1. Principles and Models

Based on random finite set theory, this model integrates diverse information in unfamiliar environments and constructs a random finite set model of map features and observational data, thereby effectively addressing data correlation challenges in complex settings.

### 2.2. RFS Models

Both the robot’s map collection and the global map collection gradually expand throughout the SLAM process as the robot travels constantly and creates maps.

$X_{k}=\left\{{x_{k}}^{1},{x_{k}}^{2},\cdots ,{x_{k}}^{N_{k}}\right\}$ is the RFS model for all map features obtained at time step *k*. The number of map features acquired at time step *k* is indicated by $N_{k}$. Thus, the RFS model may be further described as follows for the entire collection of map features that the robot has collected over all time steps:

(1) $$X_{0:k}=X_{k}\cup \left(FoV\left(\xi_{k}\right)\cap {\bar{X}}_{0:k-1}\right)$$

In this expression, $\xi_{k}$ denotes the robot’s pose at the *k*-th time step. The term $X_{0:k}$ refers to the collection of map features, modeled as a random finite set, that have been accumulated from time step 0 up to step *k*. Meanwhile, $X_{0:k-1}$ represents the set of map features that were obtained earlier, specifically before the *k*-th time step. The notation $\mathrm{FoV}(\xi_{k})$ indicates the robot’s field of view corresponding to its pose at step *k*. Lastly, ${\bar{X}}_{0:k-1}$ is defined as the group of map features that remain unacquired until just before time step *k*.

Consequently, the random finite set corresponding to freshly acquired map features can be thought of as $\mathrm{FoV}\left(\xi_{k}\right)\cap {\bar{X}}_{0:k-1}$.

The robot’s observation RFS model is written as $Z_{k}=\left\{z_{k}^{1},z_{k}^{2},\cdots ,z_{k}^{M_{k}}\right\}$, where $Z_{k}$ is the set of observations the robot made at time step *k*, and $\left\{z_{k}^{1},z_{k}^{2},\cdots ,z_{k}^{M_{k}}\right\}$ is the full observation ensemble for the $M_{k}$ map features occurring at discrete time *k*.

Given that real-world observations are often corrupted by noise, the corresponding random finite set (RFS) can be expressed as follows:

(2) $$Z_{k}=\underset{x\in X_{k}}{⋃}\;D_{k}\left(x,\xi_{k}\right)⋃C_{k}\left(\xi_{k}\right)$$

where $C_{k}\left(\xi_{k}\right)$ represents the noise observation generated at pose $\xi_{k}$, and $D_{k}\left(x,\xi_{k}\right)$ represents the observation of the true map feature at pose $\xi_{k}$.

Within the RFS framework, the SLAM problem can be expressed as estimating the joint posterior density of the map features $X_{k}$ and the robot trajectory $\xi_{1:k}$ conditioned on the observation set $Z_{k}$. This formulation allows the robot pose and the map to be inferred simultaneously. The corresponding representation is given in Equation (3).

(3) $$\pi_{k|k}\left(X_{k},\xi_{1:k}|Z_{1:k},u_{1:k},\xi_{0}\right)=\frac{g_{k}\left(Z_{k}|X_{k},\xi_{k}\right)p_{k|k-1}\left(X_{k},\xi_{1:k}\right)}{\;\int \int \;g_{k}\left(Z_{k}|X_{k},\xi_{k}\right)p_{k|k-1}\left(\xi_{1:k},X_{k}\right)d\xi_{k}\sigma X_{k}}$$

Due to the uncertainty in unknown environments, the true robot pose is not directly accessible during the SLAM process. As a result, the joint posterior density of $X_{k}$ and $\xi_{1:k}$ cannot be computed in a straightforward manner. Instead, $\pi_{k|k}$ is factorized into two components based on conditional probability. The resulting form of the decomposed joint posterior is presented in Equation (4).

(4) $$\pi_{k}\left(X_{k},\xi_{1:k}|Z_{0:k},u_{0:k-1},\xi_{0:k}\right)=\pi_{k}\left(\xi_{1:k}|Z_{0:k},u_{0:k-1},\xi_{0}\right)\pi_{k}\left(X_{k}|Z_{0:k},\xi_{0:k}\right)$$

In this formulation, $\pi_{k}\left(\xi_{1:k}|Z_{0:k},u_{0:k-1},\xi_{0}\right)$ denotes the posterior probability distribution for the estimated pose of the robot at the *k*-th time step. The term $u_{0:k-1}$ refers to the sequence of control commands issued to the robot from time step 0 up to step k−1. Meanwhile, $\pi_{k}\left(X_{k}|Z_{0:k},\xi_{0:k}\right)$ gives the posterior probability distribution for the robot’s estimate of the map features at time step *k*.

This paper employs a Poisson Multi-Bernoulli (PMB) Filter for map feature estimation. Its fundamental approach, within the RFS Bayesian framework, simultaneously represents the posterior distribution of the map feature set as two components: “undetected features” and “detected features.” The former characterizes potential features that have not yet been observed but may exist, while the latter characterizes observed feature components that are continuously maintained. Compared to PHD-based methods that approximate the target set solely by intensity, the PMB representation explicitly captures both the existence uncertainty and quantity uncertainty of features. This enables more robust handling of missed detections, false alarms, and noise interference, thereby improving the accuracy of feature quantity and location estimation in the map.

Under the PMB representation, the map feature set (conditional on the robot’s pose) consists of two types of finite random sets: the undetected feature set is modeled by a Poisson point process (PPP), whose statistical properties are described by the PPP intensity function; the detected feature set is modeled by a multi-Bernoulli (MB) process, where each Bernoulli component contains the existence probability and state probability density of that feature. Thus, the map estimation process in SLAM is fundamentally a recursive “predict-update” process for PMB parameters (PPP intensity + MB Bernoulli parameter set).

Accordingly, given that the robot’s pose at time step k−1 is $\xi_{k-1}$ and the cumulative observations are $Z_{0:k-1}$, the posterior distribution of the map features PMB at time step k−1 can be represented by the following parameter set:

(5) $$\pi_{k-1}\left(X_{k-1}|Z_{0:k-1},\xi_{0:k-1}\right)=\left\{\xi_{k-1},{\left(\lambda \right.}_{k-1}\left(x|\xi_{k-1}\right),\{\left(r_{k-1}^{\left(l\right)},p_{k-1}^{\left(l\right)}\left(x|\xi_{k-1}\right)\right)\left.{\}}_{l=1}^{L_{k-1}}\right)\right\}$$

where $\lambda_{k-1}\left(x|\xi_{k-1}\right)$ represents the Poisson intensity function describing undetected map features; ${\left\{\left(r_{k-1}^{\left(l\right)},p_{k-1}^{\left(l\right)}\left(x|\xi_{k-1}\right)\right)\right\}}_{l=1}^{L_{k-1}}$ represents the parameter set for the multi-Bernoulli component: the *l*th Bernoulli component describes a detected map feature with probability $r_{k-1}^{\left(l\right)}$ and conditional probability density $p_{k-1}^{\left(l\right)}\left(x|\xi_{k-1}\right)$; $L_{k-1}$ represents the number of Bernoulli components at time k−1.

### 2.3. Graph Optimization

As outlined earlier, the estimation of both the map and the robot’s pose forms a probabilistic inference problem that is inherently coupled yet still separable. Despite the adoption of mainstream RFS-based approaches for SLAM, a number of difficulties persist when operating within complex environments. First, when employing Rao-Blackwellized (RB) particle filtering for robot pose estimation, many particles are typically required to ensure estimation accuracy, thereby compromising the algorithm’s real-time performance. Second, the recursive estimation process has limited capacity to utilize historical information, making estimates susceptible to cumulative errors and consequently degrading localization accuracy.

As the robot continues to move and accumulates environmental observations, errors in both pose and map estimation propagate and accumulate progressively. Once a localization or mapping deviation occurs, the error amplifies continuously in subsequent time steps, potentially leading to SLAM failure in severe cases. To enhance positioning stability and accuracy, this paper introduces an Information-Controlled Pose Graph Constraint Selection Mechanism (IC-PGCS) within the Poisson Multi-Bernoulli (PMB) filtering framework. This mechanism evaluates constraint validity using distance thresholds and information control quantities, integrating optimization principles into the recursive estimation process. This approach effectively utilizes historical estimation information to perform consistent corrections on the robot’s pose, thereby suppressing long-term cumulative errors and enhancing system robustness.

Observational constraints can be expressed as

(6) $${\left(z_{k}^{vo}-h\left(\zeta_{k},X_{k}\right)\right)}^{T}Q_{k}^{-1}\left(z_{k}^{vo}-h\left(\zeta_{k},X_{k}\right)\right)$$

In this expression, $z_{k}^{vo}$ denotes the actual observation of the real landmark, while $Q_{k}$ is the covariance matrix associated with the measurement noise. The function $h(\zeta_{k},X_{k})$ corresponds to the observation model. From this constraint, the resulting cost function can be derived as follows.

(7) $$e_{k,z,vo}=z_{k}^{vo}-h\left(\zeta_{k},X_{k}\right)$$

The constraint governing motion can be stated as

(8) $${\left(\zeta_{k}-g\left(u_{k},\zeta_{k-1}\right)\right)}^{T}R_{k}^{-1}\left(\zeta_{k}-g\left(u_{k},\zeta_{k-1}\right)\right)$$

where $R_{k}$ denotes the covariance matrix of motion noise, and $g(u_{k},\zeta_{k-1})$ represents the robot’s motion model. The cost function obtained from this constraint takes the form:

(9) $$e_{k,f}=\zeta_{k}-g\left(u_{k},\zeta_{k-1}\right)$$

## 3. Methods

In this section, we present the overall architecture and the integrated SLAM system.

### 3.1. Structure of GB-PMB-SLAM Method

The overall workflow of the GB-PMB-SLAM framework consists of two main components: map feature estimation and robot pose estimation. During the phase of map estimation, the PMB-SLAM technique is employed to infer the state of the map, whereas the robot’s pose is determined using a method based on graph optimization. In contrast to the Rao–Blackwellized Particle Filter (RBPF), the proposed GB-PMB-SLAM framework lessens the need for a large number of particles when approximating the posterior distribution of the robot’s state. Rather, the estimation of the robot’s pose is cast within a graph optimization formulation, leading to enhanced computational efficiency and better consistency in estimation. In the map estimation process, undetected landmarks are represented by PPP components. During the update step, each measurement is compared against all predicted PPP components, and the likelihood-weighted PPP intensity is accumulated. When this accumulated likelihood exceeds a predefined threshold, the measurement is converted into a new Bernoulli component, whose existence probability is computed as the normalized ratio between the PPP-originated likelihood and the sum of clutter intensity and PPP-originated likelihood. In addition, the optimization module alleviates the complexity associated with explicit data association by constructing constraints based on probabilistic feature representations, thereby helping maintain target consistency without requiring exhaustive matching procedures or complicated association strategies.

In contrast to conventional graph optimization techniques, the Information-Controlled Pose Graph Constraint Selection (IC-PGCS) in the PMB-SLAM method adopts a unique approach for forming observation constraint edges. Rather than directly linking the robot to actual map features, the IC-PGCS Poisson-Bernoulli filtering SLAM method builds observation edges based on the map features estimated by the robot after performing PMB filtering. Consequently, the errors arising from this optimization procedure remain very small. This implies that observation edges that correspond to an identical map feature at different time instants are considered to have a high degree of reliability.

**Figure 1:**
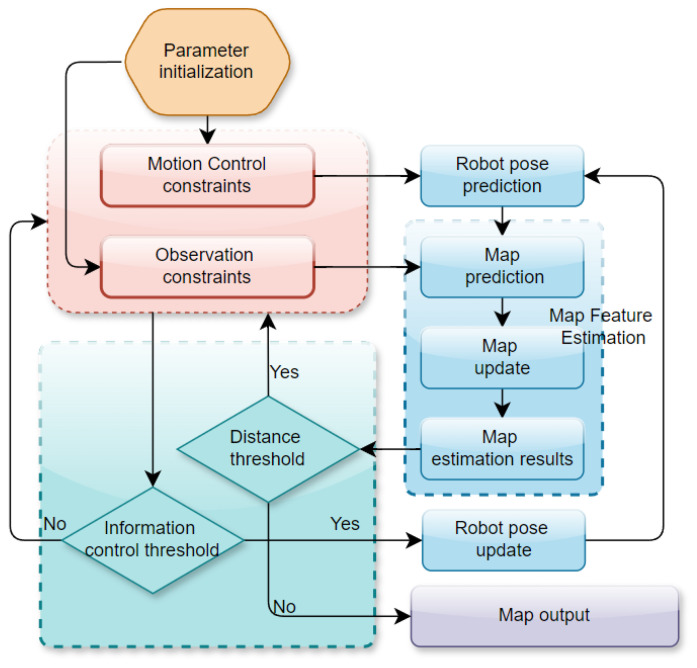
Structure block diagram of GB-PMB-SLAM system.

As illustrated in Figure 1, the proposed method follows a staged processing pipeline. Initially, the robot predicts its pose using reliable initial conditions and an accurate motion model. Based on this predicted pose, landmark prediction and estimation are subsequently carried out. Afterward, previously estimated robot states from different time steps that meet specific criteria are incorporated into the current landmark update procedure, allowing for refined estimation at the current time step. The IC-PGCS filtering strategy is then applied to determine the size of the optimization window, within which pose optimization is performed. In this framework, the nodes of the graph correspond to both the estimated landmark positions and the robot’s poses associated with the chosen time steps inside the optimization window. Meanwhile, the edges capture constraints arising from motion as well as those related to observations. Ultimately, the final landmark estimates are combined to generate the full SLAM result.

### 3.2. Map Feature Estimation

At time step *k*, assuming the robot pose is given by $\xi_{k}$ predicted by the IC-PGCS, and assuming that in the posterior distribution of the previous time step, the posterior Gaussian mixture density for the Poisson component intensity is

(10) $$\lambda_{k}^{u}\left(x|Z_{k-1}\right)=\sum_{j=1}^{J_{k}^{u}}w_{k}^{u}\left(j\right)N\left(x;\mu_{k}^{u}\left(j\right),P_{k}^{u}\left(j\right)\right)$$

where $w_{k}^{u}\left(j\right)$ represents the posterior weight of the *j*th target, $\mu_{k}^{u}\left(j\right)$ represents the Poisson prior mean of the *j*th target, $P_{k}^{u}\left(j\right)$ represents the Poisson prior covariance of the *j*th target, *u* represents the posterior Poisson intensity, and $J_{k}^{u}$ denotes the number of targets expressed as Poisson intensity.

The robot incorporates the pruned and merged Gaussian component information into the new map feature prior at time step *k*, forming the prior information set at time step *k*. The new target strength of the robot’s new map feature $b(x|\xi_{k})$ is defined as

(11) $$\lambda_{k}^{b}\left(x|Z_{k-1},M_{k-t:k}\right)=\sum_{j=1}^{J_{k}^{b}}w_{k}^{b}\left(j\right)N\left(x;\mu_{k}^{b}\left(j\right),P_{k}^{b}\left(j\right)\right)$$

where $w_{k}^{b}\left(j\right)$ represents the weight of the *j*th new target, $\mu_{k}^{b}\left(j\right)$ represents the Poisson mean of the *j*th new target, $P_{k}^{b}\left(j\right)$ represents the Poisson covariance of the *j*th new target, $J_{k}^{b}$ represents the number of new targets formed, $M_{k-t:k}$ represents the estimated map features obtained during the previous optimization iteration; it is included as a conditioning variable in the birth intensity to indicate that the previous optimization results influence the prediction of new map features. The variable *t* represents the cumulative number of time steps within the optimization window.

Assuming survival probability $P_{S}$ is constant, the transition density function f(x|y)=N(x:Fy,Q) is defined where *F* represents the state transition matrix and *Q* represents the noise matrix. Then, based on the known results of the Kalman filter prediction step from Reference [30], the Poisson partial predicted intensity we obtain is a Gaussian mixture form,

(12) $$\lambda_{k|k-1}\left(x|\xi_{k|k-1}\right)=\lambda_{k}^{b}\left(x|Z_{k-1},M_{k-t:k}\right)+P_{S}\sum_{j=1}^{J_{k}^{u}}w_{k}^{u}\left(j\right)N\left(x;F\mu_{k}^{u}\left(j\right),FP_{k}^{u}\left(j\right)F^{T}+Q\right)$$

where $F^{T}$ denotes the transpose of *F*.

At time step k−1, the robot’s pose $\xi_{k-1}$ can be obtained through motion model and pose map optimization. At this point, the set of detected map features corresponding to the set of Bernoulli terms is written as

(13) $$\pi_{k-1}={\left\{\left(r_{p,k-1}^{l},p_{p,k-1}^{l}\left(x|\xi_{k-1}\right)\right)\right\}}_{l=1}^{L_{k-1}}$$

where $p_{p,k-1}^{l}\left(x|\xi_{k-1}\right)$ is expressed as a Gaussian mixture model,

(14) $$p_{p,k-1}^{l}\left(x|\xi_{k-1}\right)=\sum_{j=1}^{J_{k-1}^{l}}w_{k-1}^{l}\left(j\right)N\left(x;\mu_{k-1}^{l}\left(j\right),P_{k-1}^{l}\left(j\right)\right)$$

In the equation, $w_{k-1}^{l}\left(j\right)$, $\mu_{k-1}^{l}\left(j\right)$, and $P_{k-1}^{l}\left(j\right)$ represent the weight, mean, and covariance matrix of the *j*th Gaussian component of the *l*th multi-Bernoulli term, respectively; $J_{k-1}^{l}$ denotes the number of Gaussian components corresponding to the *l*th multi-Bernoulli term.

For the multi-Bernoulli terms of the detected map features, propagate the existence probability and state density during the prediction phase,

(15) $$r_{p,k|k-1}^{l}=r_{p,k-1}^{l}p_{S,k}$$

(16) $$p_{p,k|k-1}^{l}\left(x|\xi_{k|k-1}\right)=\sum_{j=1}^{J_{k|k-1}^{l}}w_{k|k-1}^{l}\left(j\right)N\left(x;\mu_{k|k-1}^{l}\left(j\right),P_{k|k-1}^{l}\left(j\right)\right)$$

Thus, the prior for predicting the map feature at time k retains the PMB structure and can be expressed as

(17) $$\pi_{k|k-1}=\left(\lambda_{k|k-1}\left(x|\xi_{k}\right),{\left\{\left(r_{p,k|k-1}^{l},p_{p,k|k-1}^{l}\left(x|\xi_{k-1}\right)\right)\right\}}_{l=1}^{L_{k|k-1}}\right)$$

Upon obtaining the observation set $Z_{k}$ at time *k*, the prediction prior is updated using the detection probability $P_{D}\left(\cdot \right)$ and the measurement likelihood ℓ(·). The PMB filtering update procedure consists of three components: updating the Poisson component, updating the first detected latent detection target, and updating the surviving targets.

(18) $$\lambda_{k}\left(x|\xi_{k}\right)=\left(1-P_{D}\left(x|\xi_{k}\right)\right)\lambda_{k|k-1}\left(x|\xi_{k}\right)$$

For targets that remain alive, each single-target hypothesis can be covered as either an update using a single measurement or a missed detection. The Bernoulli parameter corresponding to the trajectory update of the single-target hypothesis is

(19) $$r_{p,k}^{l}=1$$

(20) $$p_{p,k}^{l}\left(x|\xi_{k}\right)=\frac{\sum_{j=1}^{J_{k|k-1}^{l}}w_{k|k-1}^{l}\left(j\right)N\left(x;\mu_{k|k-1}^{l}\left(j\right),P_{k|k-1}^{l}\left(j\right)\right)\ell \left(x\right)}{\sum_{l=1}^{L_{k|k-1}}\sum_{j=1}^{J_{k|k-1}^{l}}w_{k|k-1}^{l}\left(j\right)N\left(l;\mu_{k|k-1}^{l}\left(j\right),P_{k|k-1}^{l}\left(j\right)\right)\ell \left(l\right)}$$

where the existence probability is set to 1 conditioned on the successful association of a measurement to this Bernoulli component. where ℓ(·) corresponds to the likelihood function associated with each measurement.

For the *l*th multi-Bernoulli term among detected map features, the probability of its existence is updated as follows in the case of a missed detection,

(21) $$r_{p,k}^{l}=\frac{r_{p,k|k-1}^{l}\left(1-p_{D}\right)}{1-r_{p,k|k-1}^{l}+r_{p,k|k-1}^{l}\left(1-p_{D}\right)}$$

Its state density maintains the predictive form under missed detection updates as follows,

(22) $$p_{p,k}^{l}\left(x|\xi_{k}\right)=p_{p,k|k-1}^{l}\left(x|\xi_{k|k-1}\right)$$

For updates to potential detection targets detected for the first time, the corresponding Bernoulli parameter is

(23) $$r_{p,k}^{l}=\frac{\sum_{l=1}^{L_{k|k-1}}\lambda_{k|k-1}\left(l|\xi_{k|k-1}\right)\;\ell \left(l\right)}{\sum_{l=1}^{L_{k|k-1}}\lambda_{k|k-1}\left(l|\xi_{k|k-1}\right)\;\ell \left(l\right)\;+\;\kappa \left(z\right)}$$

(24) $$p_{k}^{l}\left(x,\xi_{k}\right)=\frac{\ell \left(x\right)\lambda_{k|k-1}\left(x|\xi_{k|k-1}\right)}{\sum_{l=1}^{L_{k|k-1}}\lambda_{k|k-1}\left(l|\xi_{k|k-1}\right)\ell \left(l\right)}$$

As time progresses, the computational burden of the algorithm increases with the growing number of Gaussian terms in the Poisson component and Bernoulli terms in the MB component. To address this issue, this paper employs a pruning strategy. Specifically, thresholds $T_{\lambda }$ and $T_{B}$ are set for the Poisson and Bernoulli components, respectively. Gaussian terms and Bernoulli components with weights below these thresholds are then removed. The value of $T_{B}$ can be selected by referring to multi-Bernoulli methods, while $T_{\lambda }$ must be kept at a low level to ensure the algorithm operates effectively. After pruning, map features must be extracted, primarily involving the estimation of the number of feature points and their locations. A feature extraction threshold *T* is set, and Gaussian components with weights greater than *T* after pruning and merging are extracted; the number of map features is equal to the number of Gaussian components with weights greater than *T*, and the mean of each Gaussian component corresponds to the feature’s location on the map. To further improve the accuracy of the position estimates, the weighted average of all Gaussian components is ultimately used as the actual position of the map feature.

### 3.3. Robot Pose Estimation

Once environmental descriptors are extracted at each temporal step, optimization of the pose graph is subsequently conducted. Since identical environmental points may appear multiple times at different timestamps, and because these points are recursively estimated using the PMB filter, observation connections can then be established based on the predicted descriptors. Because the observation constraints are derived from the filtered feature estimates, the associated noise can be considered relatively small. Meanwhile, motion constraints are incorporated by introducing denoised motion edges, enabling the extraction of reliable motion information. With the vertex and edge information defined, a pose graph corresponding to the same feature estimates can be constructed. Subsequently, an optimization process is employed to estimate the robot state that most effectively fulfills the defined edge constraints.

After the optimization step, a set of robot pose estimates corresponding to different time instances within the interval is obtained. These estimates are then evaluated against the original pose results, and the pose associated with the optimal state is selected for use in the map feature estimation at the subsequent time step. The selected pose is further utilized as the reference state for the PMB prediction stage, serving as the initial estimate for the robot pose in the next time step. Combined with the motion model, it provides the necessary pose information to support the following map feature estimation process.

The observation equation is given by Equation (25),

(25) $$e_{k,z}=z_{k}-h\left(\zeta_{k}\right)$$

In this setting, since the observations are constructed based on estimated map features, the resulting observation residual $e_{k,z}$ can be considered relatively small. Experimental findings suggest that the relative pose between the robot and environmental features can be directly reconstructed using triangulation, thus minimizing the reliance on a substantial number of particles commonly needed in traditional RFS-based SLAM approaches for posterior estimation. As a result, the proposed approach improves computational efficiency and supports more effective real-time performance in single-robot SLAM applications.

The distance-to-gate threshold is given by Equation (26),

(26) $$d_{gate}=\rho +v\times \Delta t\;$$

**Figure 2:**
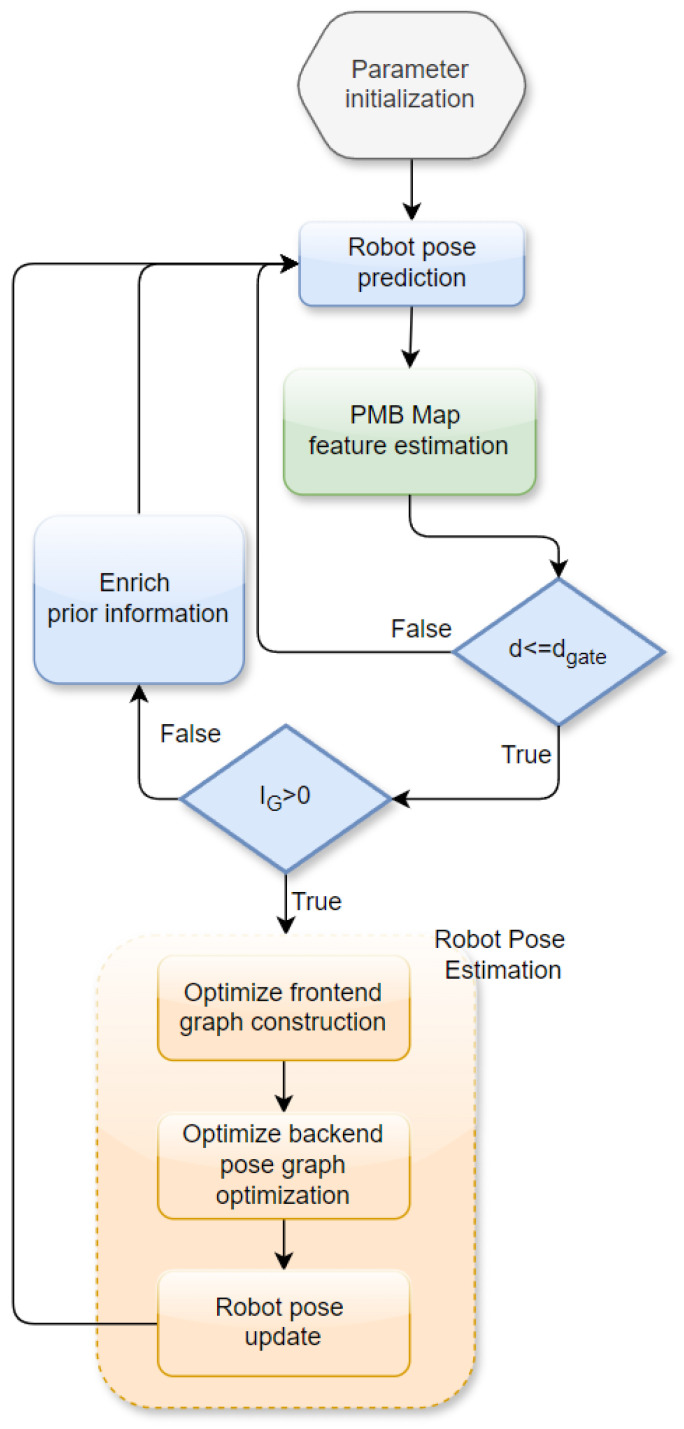
The structure of the IC-PGCS method.

The information control quantity is given by Equation (27),

(27) $$I_{CS}=\ln\left(\frac{\left(N_{f}\left(\xi_{j}\right)-N_{f}\left(\xi_{i}\right)\right)+\sum_{j=i}^{n_{f}}N_{z}\left(\xi_{j}\right)\;}{I_{\min}}\right)$$

where $N_{z}\left(\xi_{j}\right)\;$ represents the number of map features observed when the robot’s pose is $\xi_{j}$, $N_{f}\left(\xi \right)$ represents the number of edges formed by motion information, $I_{CS}$ is the information control value, and $I_{\min}$ is the adaptive information control threshold. When $I_{CS}\leq 0$, proceed to the next step of PMB to estimate the map feature state. Set k=k+1,t=t+1. Add the Gaussian components corresponding to the map features obtained at the previous time step to the newly generated map set in the next map feature estimation process to enrich the prior information (as shown in Equation (11)). When $I_{CS}>0$, perform backend optimization on the robot poses at time steps *t* to obtain and update the optimal poses corresponding to these time steps, then set t=0. The structure of the IC-PGCS method is shown in Figure 2.

The observation constraints are constructed as follows. First, raw radar measurements are used in the PMB update. Bernoulli components with existence probability above a threshold are extracted as probabilistic feature hypotheses. Second, candidate observation edges are generated only when the predicted feature hypotheses satisfy the distance gate given by Equation (26). The covariance of each observation edge is computed by combining the sensor measurement covariance and the posterior covariance of the corresponding Bernoulli component. Hence, the resulting residuals are not assumed to be zero; they reflect both measurement and estimation uncertainties.

The computational complexity of the PMB filter update is $O\left((J_{\mathrm{PPP}}+J_{\mathrm{MB}})\cdot M\right)$, where $J_{\mathrm{PPP}}$ and $J_{\mathrm{MB}}$ are the numbers of PPP and Bernoulli Gaussian components, respectively, and *M* is the number of measurements per time step. The IC-PGCS module triggers pose graph optimization only when the information-control score exceeds $I_{\min}$; therefore, the average complexity per step remains O(K) for the number of pose graph nodes in the sliding window.

The PMB-SLAM implementation with Pose Graph Optimization Triggered by information-control score is shown in Algorithm 1.
**Algorithm 1** GB-PMB-SLAM Module with IC-PGCS  1:**Input:** odometry measurement $u_{k}$, observation set $Z_{k}$  2:**Output:** robot pose estimate $\xi_{k}$, map feature set $X_{k}$  3:Initialize robot pose $\xi_{0}$  4:Initialize map RFS $\left(\lambda_{0}\left(x\right),{\left\{\left(r_{0}^{\left(l\right)},p_{0}^{\left(l\right)}\left(x\right)\right)\right\}}_{l=1}^{L_{0}}\right)$  5:Set optimization window length t←0  6:**for** k=1 to *K* **do**  7:      Predict robot pose $\xi_{k|k-1}=g\left(u_{k},\xi_{k-1}\right)$  8:      Predict Poisson intensity $\lambda_{k|k-1}\left(x|\xi_{k|k-1}\right)$(Equation (12))  9:      Predict Bernoulli parameters $\left(r_{p,k|k-1}^{l},p_{p,k|k-1}^{l}\left(x|\xi_{k|k-1}\right)\right)$(Equations (15) and (16))10:      Update Poisson component $\lambda_{k}\left(x|\xi_{k}\right)$(Equation (18))11:      Update Bernoulli components $\left(r_{p,k}^{l},p_{p,k}^{l}\left(x|\xi_{k}\right)\right)$(Equations (19) and (20))12:      Perform Gaussian pruning and merging13:      Extract candidate map feature hypotheses from Bernoulli components14:      Compute the distance gate threshold $d_{\mathrm{gate}}=\rho +v\Delta t$(Equation (26))15:      Compute the distance *d* between the current feature hypothesis and the candidate historical feature hypothesis16:      **if** $d\leq d_{\mathrm{gate}}$ **then**17:            Generate candidate observation constraints within the distance gate18:            Compute information-control score $I_{\mathrm{CS}}$(Equation (27))19:            **if** $I_{\mathrm{CS}}>0$ **then**20:                 Construct frontend pose graph G=(V,E)21:                 Add motion constraints to *E*22:                 Add gated observation constraints to *E*23:                 Perform backend pose graph optimization in the sliding window24:                 Update robot pose estimate $\xi_{k}$25:                 t←026:            **else**27:                 Enrich the prior information using map features estimated in the current window28:                 t←t+129:            **end if**30:      **else**31:            Reject the candidate observation constraints outside the distance gate32:            t←t+133:      **end if**34:      Extract final map feature estimates $X_{k}$ from Bernoulli components35:**end for**36:**return** $\xi_{k}$, $X_{k}$

## 4. Results

In this section, the experimental results are presented and analyzed. Both simulation experiments and real-world experiments are conducted to comprehensively evaluate the proposed method. The comparison methods include:

1. RB-PHD-SLAM, a first-order moment approximation method based on Random Finite Sets (RFS), where the robot pose is estimated using particle approximation;
2. MBer-SLAM, which employs a Multi-Bernoulli filtering framework for joint map and pose estimation;
3. GB-CBMber-SLAM, a reference method that adopts Cardinality Balanced Multi-Bernoulli (CBMber) map estimation combined with AIC-triggered pose graph optimization;
4. GB-PMB-SLAM, which upgrades the map estimation to a Poisson Multi-Bernoulli (PMB) framework and incorporates an information-controlled pose graph constraint selection mechanism (IC-PGCS).

The effectiveness and robustness of the proposed method are validated through comprehensive comparative analysis with these approaches.

### 4.1. Simulation Experiments

The simulation environment runs on the MATLAB 2024b platform, with a map area of 120 m × 400 m comprising 19 coordinates. The robot’s sensor is a millimeter-wave radar. Gaussian noise is employed throughout the simulation experiments. Simulation environment parameters are detailed in Table 1. During experimentation, the proposed GB-PMB-SLAM method is compared with the aforementioned RB-PHD-SLAM, MBer-SLAM, and GB-CBMber-SLAM methods.

In the experiments, the true positions of map features are denoted by “◯”, estimated positions by “+”, and noise in the map environment by “×”. The black dashed line represents the robot’s true pose information, while the red “-□-”, light blue “-□-”, magenta “-□-”, and blue “-□-” lines respectively indicate the estimated robot poses from the four methods. The specific experimental data are shown in Table 2.

**Figure 3:**
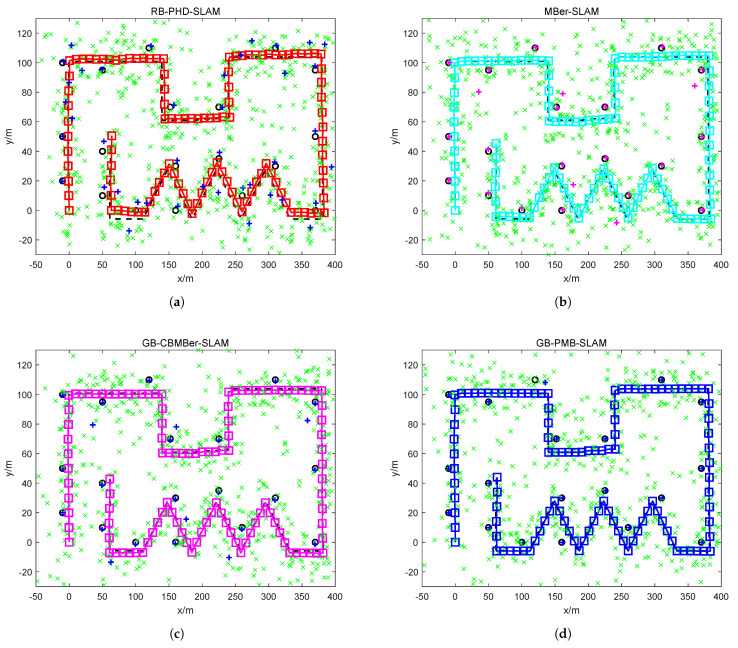
(**a**) RB-PHD-SLAM method. (**b**) MBer-SLAM method. (**c**) GB-CBMber-SLAM method. (**d**) GB-PMB-SLAM method.

As shown in Figure 3, the overall trajectory comparison of the four methods in a single Monte Carlo (MC) simulation experiment reveals that RB-PHD-SLAM exhibits significant trajectory deviation due to particle decay and noise interference. MBer-SLAM partially improves trajectory consistency. while GB-CBMber-SLAM and the proposed GB-PMB-SLAM exhibit trajectories closer to the true path overall. Among these, GB-PMB-SLAM demonstrates greater stability during continuous turns and extended operation segments. This indicates that the GB-PMB-SLAM method achieves superior overall SLAM performance compared to the other three approaches. Subsequent analysis compares robot pose error, feature count error, Optimal Subpattern Assignment (OSPA) distance, and computational time.

**Figure 4:**
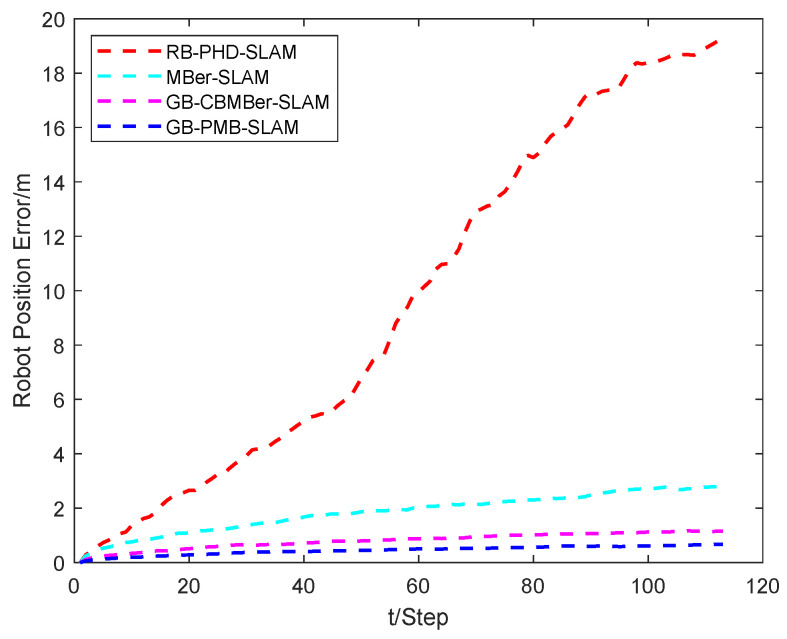
Comparison of robot pose estimation error.

As shown in Figure 4, the curves depict the temporal evolution of robot position error for the four algorithms across 100 Monte Carlo simulations. The curves reveal that RB-PHD-SLAM exhibits cumulative error over time with pronounced peaks; while MBer-SLAM’s error decreases but still shows an increase in the middle and latter stages. The two pose map-based optimization methods (GB-CBMber-SLAM and GB-PMB-SLAM) exhibit significantly lower overall errors. Further comparison reveals that GB-PMB-SLAM’s error increases more gradually with smaller fluctuations, indicating that the proposed method demonstrates better stability under complex observation conditions. This is because PMB explicitly absorbs unconfirmed features via PPP, delivering “cleaner” features to MB and reducing contamination of backend constraints by erroneous features. Concurrently, IC-PGCS selectively introduces high-value constraint edges based on information-control score, minimizing bias caused by low-information or low-reliability edges entering the pose graph.

**Figure 5:**
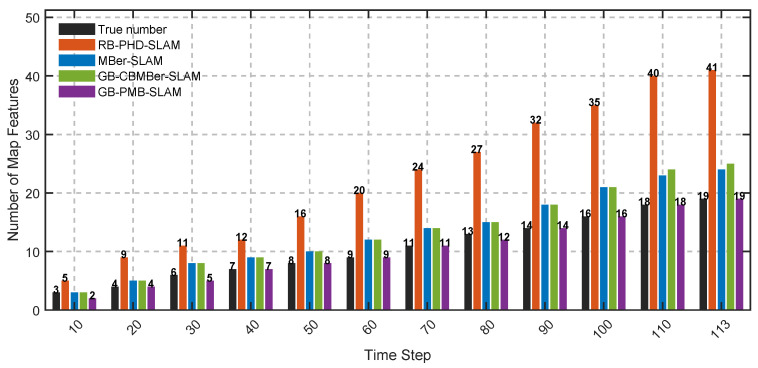
Comparison of map feature number estimation.

As shown in Figure 5, the map feature count estimates from four methods during a single Monte Carlo (MC) simulation are displayed, with the black bars representing the true feature count. RB-PHD-SLAM exhibits significant overestimation, with feature counts rapidly increasing over time and far exceeding the true value. Both MBer-SLAM and GB-CBMber-SLAM suppress overestimation, yet their estimated feature counts still deviate from the true value. GB-PMB-SLAM effectively suppresses overestimation, with its estimated feature count consistently close to the black bars. Notably, the GB-PMB-SLAM result aligns most closely with the true count, indicating that the PMB structure better controls map cardinality and avoids the accumulation of false new features in noisy environments. This occurs because PPP represents latent but unconfirmed features, generating new Bernoulli events into MB only when observations provide sufficient evidence. For persistently unconfirmed latent features, PPP decays during missed detection updates, thereby preventing infinite accumulation of latent inventory.

**Figure 6:**
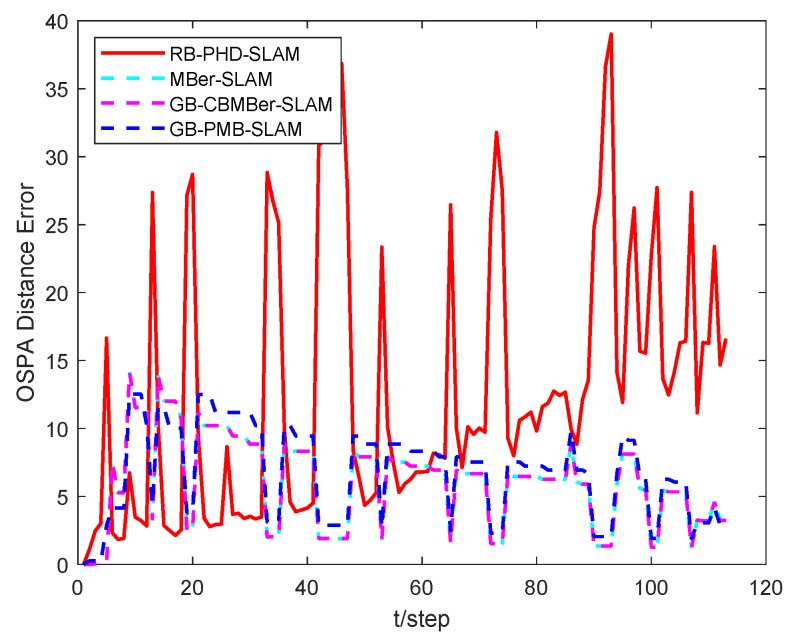
Comparison of OSPA distance error.

Figure 6 shows the OSPA distance error over time for the four methods in 100 Monte Carlo (MC) simulation experiments. In this case, the OSPA cut-off distance is set to 2, and the distance is of order 1. Lower OSPA values indicate better map estimation performance, including both localization accuracy and cardinality consistency. The RB-PHD-SLAM curve exhibits frequent spikes, indicating significant cardinality errors and positioning errors coexisting in noisy environments. The MBer-SLAM and both graph optimization methods demonstrate markedly lower and more stable OSPA errors. Among these, GB-PMB-SLAM achieves comparable OSPA performance with smaller fluctuations, reflecting the synergistic effect of PMB in suppressing false new births and IC-PGCS in selecting reliable constraints. Although its average OSPA is slightly higher than that of MBer-SLAM and GB-CBMber-SLAM in this simulation, the reduced fluctuations indicate improved stability in map consistency.

During Gaussian weight updates for nonlinear functions, RB-PHD-SLAM employs an EKF-based local linearization. Strong nonlinearity leads to larger approximation errors. The other three methods estimate and update posterior probability densities using a single parameter set, resulting in lower computational complexity and superior real-time performance.

**Figure 7:**
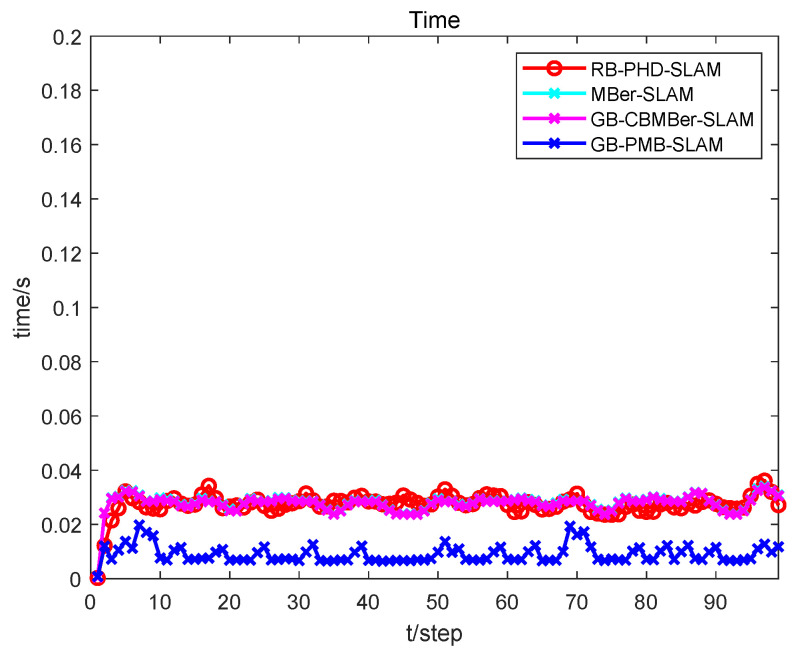
Comparison of required time.

Figure 7 compares runtime across all four methods during 100 Monte Carlo simulations. RB-PHD-SLAM exhibits the highest overall computational cost with significant fluctuations. MBer-SLAM and GB-CBMber-SLAM demonstrate markedly reduced computational demands. The proposed GB-PMB-SLAM maintains accuracy advantages while achieving superior or comparable computational efficiency. Compared to SLAM methods employing the GB-CBMber-SLAM filter, it reduces computational time by 67.9%, with overall lower computational curves. This is because PMB absorbs uncertain features through PPP, reducing maintenance and association costs caused by false features entering MB. In order to efficiently control backend computational overhead, IC-PGCS simultaneously uses information-control score to filter candidate constraint edges, avoiding low-value edges from entering the pose graph and minimizing needless optimization triggers.

**Figure 8:**
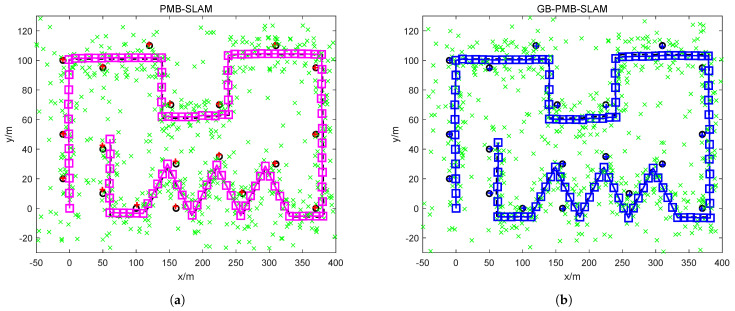
(**a**) PMB-SLAM method. (**b**) GB-PMB-SLAM method.

**Figure 9:**
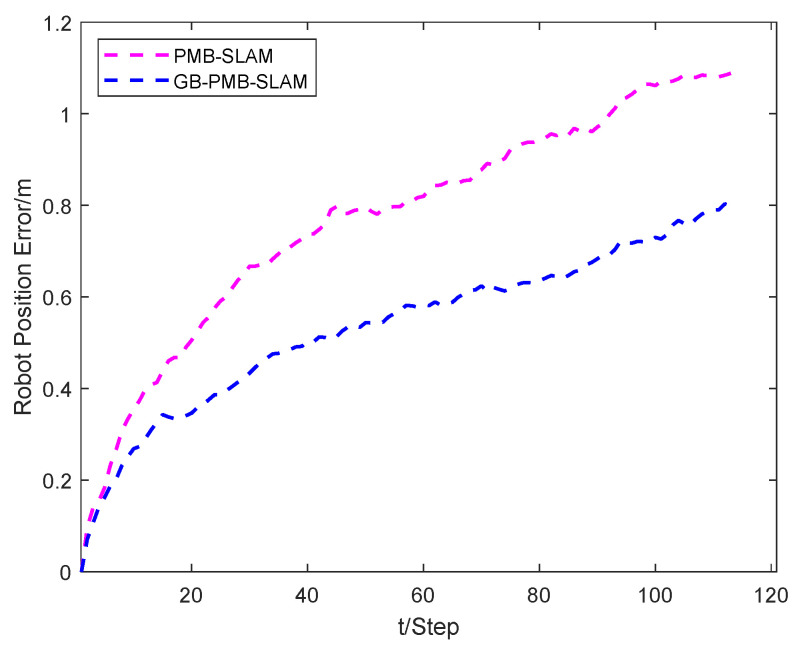
Comparison of robot pose estimation error.

**Figure 10:**
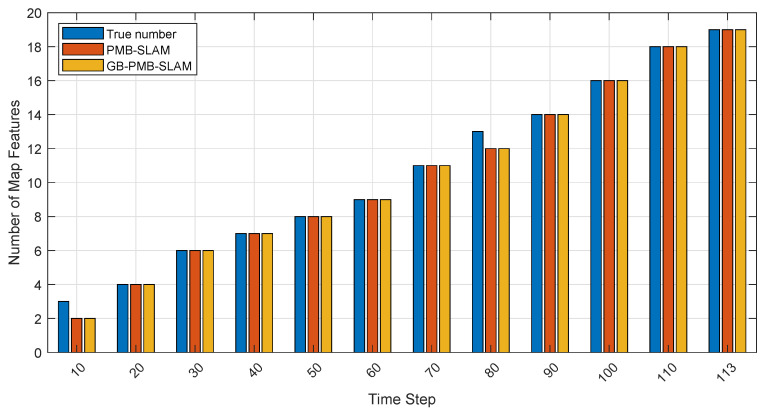
Comparison of map feature number estimation.

**Figure 11:**
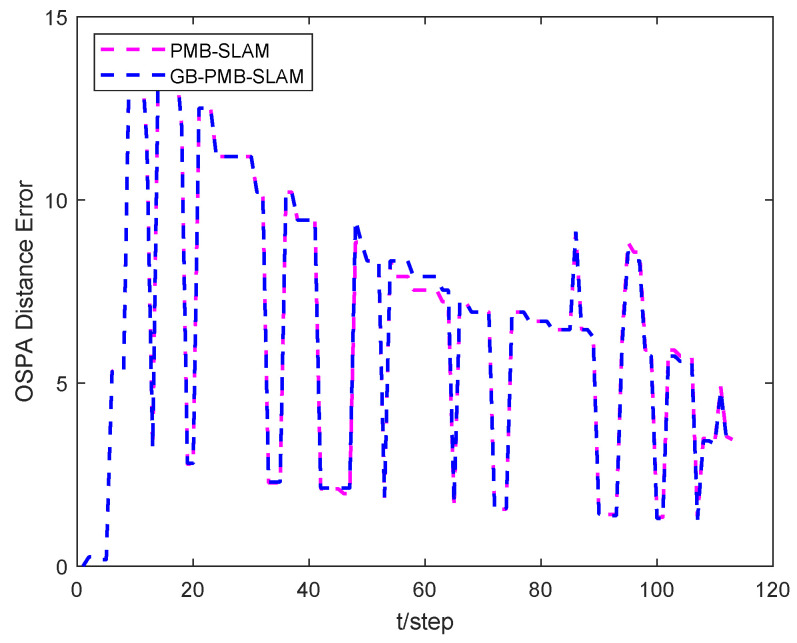
Comparison of OSPA distance error.

**Figure 12:**
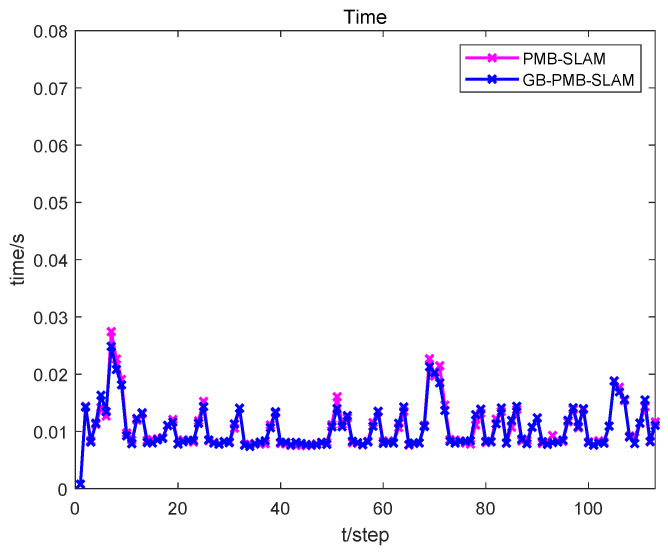
Comparison of required time.

To evaluate the contribution of the IC-PGCS module, we compare the full GB-PMB-SLAM with a variant where the IC-PGCS trigger is disabled (PMB-SLAM without pose graph constraint selection). As shown in Figure 8, the trajectory of the full GB-PMB-SLAM (blue) follows the true path more closely than the PMB-SLAM without IC-PGCS (red). Figure 9 shows that disabling IC-PGCS leads to larger pose estimation errors over time, while the full GB-PMB-SLAM maintains lower and more stable errors. In terms of map feature number estimation (Figure 10), both methods achieve accurate cardinality estimation with zero feature count error. The OSPA distance error comparison (Figure 11) shows that the two methods perform similarly, with the full GB-PMB-SLAM exhibiting slightly smaller fluctuations. Figure 12 indicates that the runtime of both methods is nearly identical, confirming that IC-PGCS does not introduce additional computational overhead.

Quantitatively, Table 3 shows that disabling IC-PGCS increases the robot position error from 0.534 m to 0.769 m, corresponding to a reduction of approximately 30.6% when IC-PGCS is enabled. The OSPA error remains nearly unchanged (6.612 m vs. 6.591 m), and the runtime is identical (0.011 s). These results indicate that the IC-PGCS module contributes to a 30.6% reduction in robot position error while maintaining nearly identical OSPA and runtime performance, confirming its effectiveness in improving localization accuracy without sacrificing map consistency or computational efficiency.

### 4.2. Real-World Experiments

**Figure 13:**
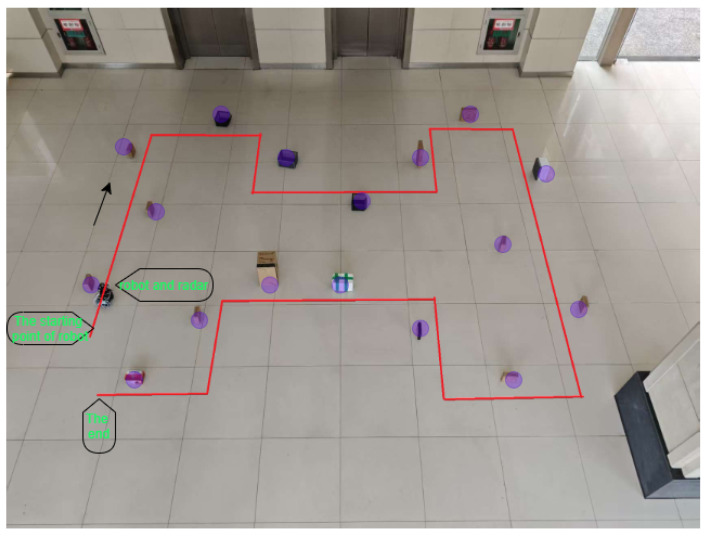
Real experimental environment.

A complex indoor setting was chosen for the experiment, with a four-wheeled cart acting as the mobile robotic platform. As the robot moved along a trajectory, it continuously perceived its environment and carried out the SLAM procedure. The data used came from actual measurements obtained using a millimeter-wave radar, which provided the observation dataset. Figure 13 depicts the real-world configuration used in the experiment.

The robot trajectory and landmark positions were manually surveyed using a Leica DISTO D2 laser rangefinder (Leica Geosystems AG, Heerbrugg, Switzerland) (accuracy: ±1.5 mm) in a predefined indoor coordinate system. These manually obtained references serve as the reference trajectory and reference map for error calculation, providing a consistent benchmark for comparing all four SLAM methods under identical conditions.

The robot platform is a four-wheeled cart equipped with wheel encoders for odometry. A Texas Instruments IWR1243 76-81 GHz FMCW millimeter-wave radar (Texas Instruments Incorporated, Dallas, TX, USA) provides range-bearing measurements with a 150° field of view and a 25 m range. Algorithms were implemented in MATLAB R2024b and executed on a host computer with an AMD Ryzen 7 7735HS CPU @ 3.2 GHz, 16 GB RAM, and an NVIDIA GeForce RTX 4060 GPU (8 GB VRAM). All reported runtimes are average computation times per time step, excluding data logging and plotting.

**Figure 14:**
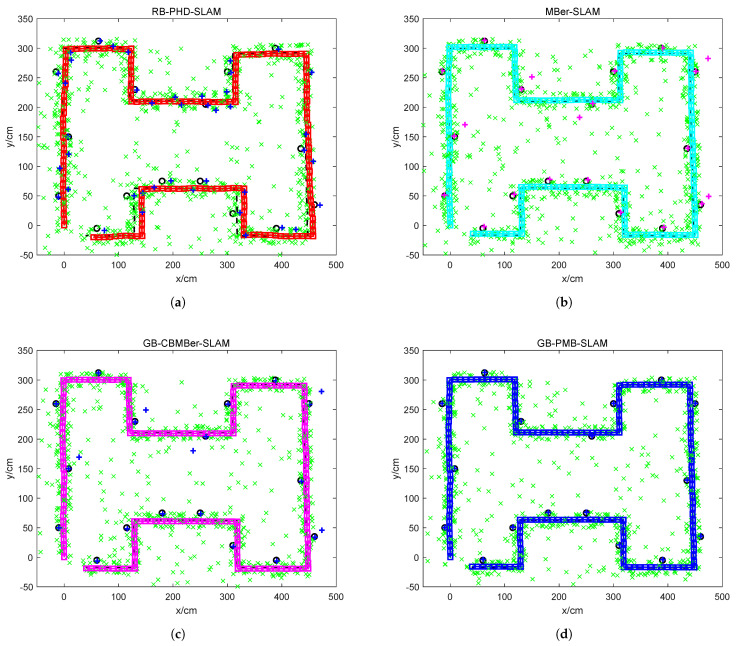
(**a**) RB-PHD-SLAM method. (**b**) MBer-SLAM method. (**c**) GB-CBMber-SLAM method. (**d**) GB-PMB-SLAM method.

Figure 14 presents the SLAM estimation results obtained from the robot using four different approaches: RB-PHD-SLAM, MBer-SLAM, GB-CBMber-SLAM, and GB-PMB-SLAM. According to Figure 14, both MBer-SLAM and GB-CBMber-SLAM achieve better performance than RB-PHD-SLAM in terms of estimating the number of map features as well as robot pose. When comparing pose estimation accuracy, GB-CBMber-SLAM surpasses MBer-SLAM. Meanwhile, GB-PMB-SLAM shows an advantage over both MBer-SLAM and GB-CBMber-SLAM in map feature count estimation, and also exceeds GB-CBMber-SLAM in pose estimation performance.

**Figure 15:**
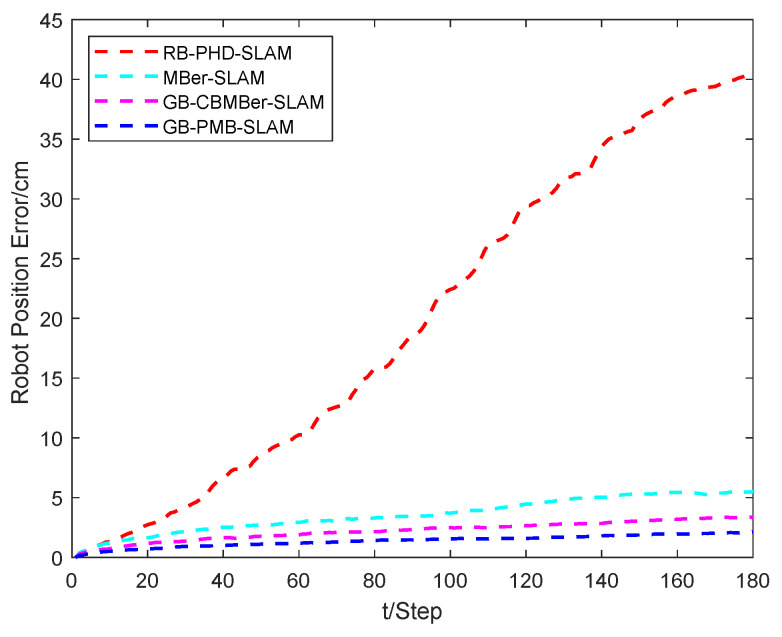
Comparison of robot pose estimation error.

The root mean square error (RMSE) of the robot’s position served as the metric for assessing localization accuracy. A comparison of the four methods was carried out by computing the pose RMSE at each individual time step. The resulting experimental outcomes are presented in Figure 15. As illustrated in Figure 15, the GB-PMB-SLAM approach demonstrates a modest advantage in pose estimation accuracy over the GB-CBMber-SLAM approach.

As can be observed from Figure 15, the estimation error associated with the RB-PHD-SLAM method grows quickly as time progresses. In contrast, the errors produced by the remaining three methods stay stable and remain at comparatively low levels. Detailed numerical results are provided in Table 4.

For assessing how well the map feature estimation performs, two comparison metrics are employed. The first involves comparing both the quantity of estimated map features at each time step and their associated pose errors against the ground truth values. The second metric is based on a comparison of the OSPA distance.

**Figure 16:**
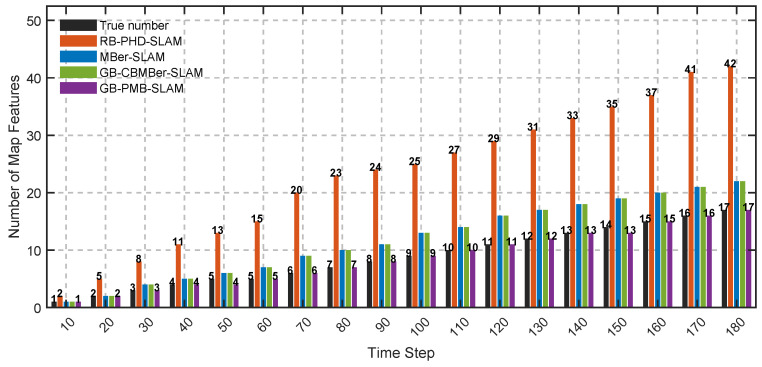
Comparison of map feature number estimation.

As illustrated in Figure 16, the quantity of map features estimated by the proposed GB-PMB-SLAM approach remains close to the actual number throughout the process.

**Figure 17:**
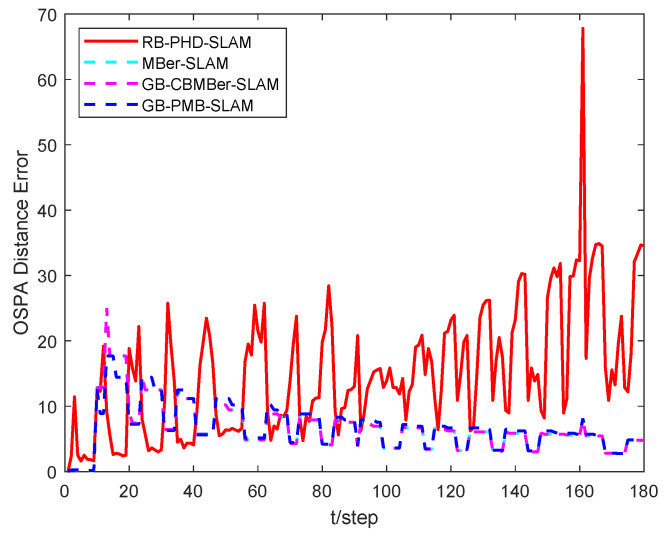
Comparison of OSPA distance error.

As shown in Figure 17, the OSPA error produced by the GB-PMB-SLAM method varies less over time compared to the RB-PHD-SLAM approach, and is comparable to those of MBer-SLAM and GB-CBMber-SLAM, although slightly higher in average value (see Table 4). This indicates that the proposed method maintains competitive map consistency while offering advantages in localization and computational efficiency.

**Figure 18:**
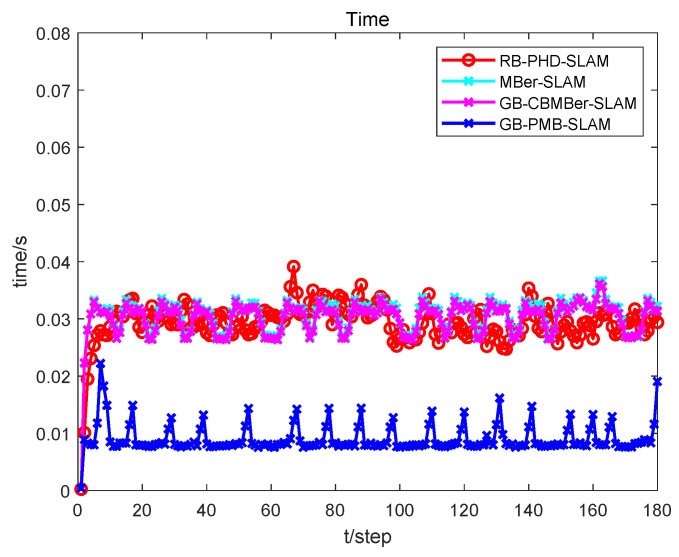
Comparison of required time.

When the GB-PMB-SLAM approach is compared against the other three methods, Figure 18 reveals that it demands substantially less computational time than its counterparts. The corresponding numerical values are provided in Table 4.

## 5. Discussion

The proposed GB-PMB-SLAM framework provides a probabilistic and information-driven solution to address key challenges in SLAM, including data association, map representation, and long-term pose estimation. By introducing the PMB formulation, the map is modeled as a unified representation consisting of undetected features (PPP) and detected features (MB), which enables consistent feature estimation and reduces the bias in feature cardinality.

The integration of pose graph optimization further improves global consistency by mitigating drift accumulation over time. In particular, the IC-PGCS strategy establishes a connection between information-control score and optimization triggering, allowing pose graph optimization to be activated only when sufficient information is available. This improves estimation accuracy while reducing redundant computations.

From an algorithmic perspective, the proposed framework avoids explicit data association by implicitly handling it within the PMB formulation, thereby maintaining a tractable recursive structure. This enables improved robustness in cluttered and feature-rich environments.

However, several limitations remain. The current formulation assumes relatively ideal sensing conditions and does not explicitly consider severe noise, occlusions, or dynamic objects, which may degrade estimation accuracy in real-world scenarios. In addition, the performance of IC-PGCS depends on the selection of the information-control score threshold $I_{m}in$; an adaptive threshold selection method will be investigated in future work. The survival probability of map features is assumed constant, which is a simplification for static environments; extending it to time-varying or object-dependent survival is a promising direction. Moreover, while the proposed method is evaluated against RFS-based SLAM approaches, a systematic comparison with modern visual or LiDAR SLAM systems (e.g., ORB-SLAM, LIO-SAM) would require different sensor configurations and is therefore beyond the scope of this paper. Such cross-modal comparisons are of interest for future work, but the present study focuses on radar-based RFS-SLAM within a consistent sensor setting. The computational cost of PMB filtering may also increase in large-scale environments, although the IC-PGCS strategy helps mitigate this by avoiding unnecessary optimizations.

Despite these limitations, the improvements in localization accuracy (Figure 4 and Table 2), feature-count estimation (Figure 5), and computational efficiency (Figure 7 and Table 2) collectively support the proposed method’s effectiveness, while the OSPA results (Figure 6) show comparable map consistency.

Furthermore, during the processing of Gaussian components, techniques such as the Unscented Kalman Filter (UKF), the Cubature Kalman Filter (CKF), and their respective extensions can be utilized to obtain the weights, means, and covariances of Gaussian components in a more robust manner. This capability allows SLAM algorithms to better accommodate unknown, evolving, and complex indoor environments. Looking ahead, artificial intelligence techniques may be integrated to optimize front-end perception and feature selection, thereby increasing the algorithm’s adaptability in unstructured settings and strengthening its robustness under highly dynamic conditions.

## 6. Conclusions

This paper proposes a pose-graph-optimization-based Poisson multi-Bernoulli SLAM method. The proposed approach reduces the reliance on explicit data association in traditional algorithms while reducing computational complexity. During the recursive process, it estimates the multi-target posterior probability density without requiring any approximate strategies for particle weight computation, thereby achieving higher accuracy and improved stability. By representing the map as a unified structure consisting of undetected features (PPP) and detected features (MB), the proposed method alleviates the bias in feature cardinality estimation in conventional SLAM algorithms and improves feature cardinality estimation.

Additionally, the IC-PGCS approach facilitates an effective integration between pose graph optimization and the PMB framework. By leveraging constraints from graph optimization, the accumulation of global errors is suppressed, which enhances long-term robustness. Experimental results show that the proposed method achieves lower robot position error and feature-count error than MBer-SLAM and GB-CBMber-SLAM, with significantly reduced runtime. In terms of OSPA error, the proposed method performs comparably to these baselines, although it is slightly higher than MBer-SLAM and GB-CBMber-SLAM in some settings (see Table 2 and Table 3). This trade-off is mainly attributed to the conservative conversion of PPP components into Bernoulli components, which helps maintain cardinality accuracy but may slightly affect spatial distribution consistency. Overall, the main advantages of the proposed method lie in localization accuracy, cardinality estimation, and computational efficiency. These findings confirm the effectiveness of the proposed method in cluttered indoor environments.

The current formulation assumes relatively ideal sensing conditions and does not explicitly consider severe noise, occlusions, or dynamic objects. The survival probability of map features is assumed constant, which may be an oversimplification in highly dynamic environments. The performance of the IC-PGCS strategy depends on the selection of the information threshold $I_{min}$; an adaptive threshold selection method would be beneficial. In future work, we plan to incorporate robust filtering techniques such as UKF or CKF to handle nonlinearities, and to integrate deep learning-based feature extraction to improve robustness in unstructured environments. Multi-robot collaborative SLAM will also be explored based on the current single-robot framework.

## Figures and Tables

**Table 1:** List of relevant parameters for robots and simulation environments.

Parameter	Value
Robot movement speed (m/s)	10
Radar scanning range (°)	150
Radar scan radius distance (m)	25
Azimuth angle observation noise (°)	1.0
Target distance observation noise (m)	0.1
Control speed noise (m)	0.3
Detection probability $P_{D}$	0.97
Survival probability $P_{S}$	0.99
Clutter intensity κ (m^−2^)	0.002 (approx. 4 clutter points within scan area)
Existence probability pruning threshold *T*	0.8
Gaussian weight pruning threshold $T_{B}$	0.01
PPP intensity pruning threshold $T_{\lambda }$	0.001
Distance gate $d_{\mathrm{gate}}$ (m)	10
Information-control score threshold $I_{\min}$	10

**Table 2:** Comparison results of simulation experiment data.

Method	Robot Position Error/m	The Number of Features in the Map	The Estimated Number Error of Features	OSPA Error/ m	Comparison of Required Time/s
RB-PHD-SLAM	9.615	19	22	12.908	0.027
MBer-SLAM	1.847	19	5	6.059	0.028
GB-CBMber-SLAM	0.804	19	5	6.059	0.028
GB-PMB-SLAM	0.451	19	0	6.785	0.009

**Table 3:** Ablation study: comparison of GB-PMB-SLAM with and without IC-PGCS.

Method	Robot Position Error/m	The Number of Features in the Map	The Estimated Number Error of Features	OSPA Error/ m	Comparison of Required Time/s
PMB-SLAM	0.769	19	0	6.591	0.011
GB-PMB-SLAM	0.534	19	0	6.612	0.011

**Table 4:** Comparison results of real-world experiment data.

Method	Robot Position Error/cm	The Number of Features in the Map	The Estimated Number Error of Features	OSPA Error/cm	Comparison of Required Time/s
RB-PHD-SLAM	19.971	19	22	15.098	0.029
MBer-SLAM	3.570	19	5	6.700	0.031
GB-CBMber-SLAM	2.218	19	5	6.692	0.030
GB-PMB-SLAM	1.380	19	0	6.830	0.009

## Data Availability

The original contributions presented in this study are included in the article. Further inquiries can be directed to the corresponding author.
